# The quality of life in children with spinal muscular atrophy: a case–control study

**DOI:** 10.1186/s12887-022-03751-y

**Published:** 2022-12-12

**Authors:** Gholamreza Zamani, Mahmoud Reza Ashrafi, Homa Ghabeli, Masood Ghahvechi Akbari, Mahmoud Mohammadi, Reza Shervin Badv, Sareh Hosseinpour, Roya Haghighi, Elham Pourbakhtyaran, Nahid Khosroshahi, Morteza Heidari

**Affiliations:** 1 Pediatrics Center of Excellence, Department of Pediatric Neurology, Children’s Medical Center, Tehran University of Medical Sciences, Tehran, Iran; 2 Department of Physical Medicine and Rehabilitation, Pediatrics Center of Excellence, Children’s Medical Center, Tehran University of Medical Sciences, Tehran, Iran; 3 Department of Pediatric Neurology, Vali-E-Asr Hospital, Imam Khomeini Hospital Complex, Tehran University of Medical Sciences, Tehran, Iran; 4 Department of Pediatric Neurology, Bahrami Children’s Hospital, Tehran University of Medical Sciences, Tehran, Iran

**Keywords:** Spinal muscular atrophy, Health-related quality of life, Quality of life, Children

## Abstract

**Objectives:**

This study aimed to analyze the health-related quality of life (HRQoL) of patients with spinal muscular atrophy (SMA) based on the type of SMA, demographic and clinical features and compare HRQoL of these patients with a matched healthy control group.

**Methods:**

This was a case–control study of Patients with SMA in Iran. Sixty-six patients with SMA type II and III aged 8–18 years and also 264 healthy age, sex, and socio-economic matched individuals were enrolled. To assess the quality of life, we used the Persian version of the KIDSCREEN-27.

**Results:**

The health-related quality of life between children with type II and type III SMA was not significant in all 5 subscales. However, HRQoL in healthy children was significantly higher than in SMA children in all 5 subscales.

**Conclusion:**

The quality of life in children with SMA was lower than the healthy control group in all subscales, and physical well-being and psychosocial aspects are the main domains of life impaired by SMA disease. However, no significant difference between the quality of life in children with SMA type II and type III was observed.

## Background

Spinal muscular atrophy (SMA) is an inherited autosomal recessive disease [1] characterized by degeneration of alpha motor neurons of the spinal cord, resulting in generalized muscle weakness and atrophy [2, 3]. SMA is the second fatal neuromuscular disease after cystic fibrosis, with an estimated incidence of about one in 6000–10,000 live births and a carrier frequency of 1/40–1/60 [4, 5].

The clinical manifestations of SMA are divided into three major categories in childhood according to the age of onset and motor function achieved [6]. The spectrum of muscle weakness in SMA ranges from extremely compromised neonates to minimally affected adults with late-onset [6, 7]. Generally, the severe form of SMA is type I which is manifested by muscle weakness and hypotonia. Children who suffer from SMA type II are able to sit, but they need aid for standing or walking; and finally, the mild form of SMA is type III who learn to walk unaided [5, 8].

Since these patients will need nursing support due to losing their motor function or respiratory compromise or nutritional problems in advanced stages [2, 9], they would be dependent on others and therefore their quality of life is touched. Since the severity and timing of these complications vary according to the type of disease [1, 10], this factor can also affect the extent of patient dependency on others. In addition, factors such as the socio-economic status of patients and the level of their access to appropriate health care can have a significant effect on their quality of life [11].

Health-related quality of life (HRQoL) is a multidimensional framework that generally evaluates social, psychological, and physical functioning of a patient [12]. Although HRQoL tools are described according to individual perspective of one’s life status along with personal judgment over patient's health and disease and also norms of the community in where they live [13], various studies indicate these tools are reliable in the assessment of standard cares that patients receive [14, 15]. There are controversy results in subscales of the quality of life in various studies and most of them do not report their results according to SMA type and demographic data [16–20]. In the current research, we aimed to answer the following questions:

First, Does HRQoL of SMA children differ from that of a matched healthy control group? Second, Does HRQoL of children with SMA vary according to SMA type? Third, Does HRQoL of SMA children differ according to demographic and clinical features?

## Methods

### Participants

We conducted a case–control study on 66 patients diagnosed with SMA aged 8–18 years who were referred to the pediatric neurology department of Tehran University of Medical Sciences where SMA patients were being monitored. We considered this age group because the patients and their parents were both adapted to the disease. We also selected 264 healthy age, sex, and socio-economic matched individuals and their parents from the same school in which patients attended as controls. The inclusion criteria were age range of 8–18 years, SMA diagnosis was done based on clinical manifestations, clinical fitures,electrodiagnosis and confirmed by a genetic test. Clinical features such as respiratory and gastrointestinal diseases were diagnosed through medical examination and obtained retrospectively from patients’ medical records. The exclusion criteria were mental disorders, hearing, or visual impairments, and or any congenital disorder. This study was approved by the ethical committee of Tehran University of Medical Sciences (IR.TUMS.CHMC.REC.1400.063), furthermore written informed consent was taken from all participants’ legal guardians collaborating in this study.

### Assessment tools and data collection

To assess the quality of life, we used the Persian version of the KIDSCREEN-27. All versions of this questionnaire, including child and parent’s versions that had been validated for Iranian population aged 8–18 years old [21]. Since mothers usually spend much more time with kids [22], the parent's version of KIDSCREEN-27 was completed mostly by mothers. In our study, the parents and children both completed the questionnaire at home. This questionnaire consists of 5 categories, including physical well-being (5 items), psychological well-being (7 items), parent relationship (7 items), social support and peers (4 items), social environment (4 items). The recall period lasted two weeks, and the higher score revealed a better quality of life.

### Statistical analysis

We described the quantitative data using mean and standard deviation (SD) and the categorical data using frequency and percentage. Moreover, we checked the normality assumption of data prior to the analysis, then we compared the health-related quality of life between the SMA types (type II and type III) and also between the case and control groups, using independent-samples T test. The health-related quality of life was also compared according to demographic and clinical features, listed in Table 1. The P-value of less than 0.05 was considered significant. All analyses were performed using SPSS version 24.

**Table 1:** Comparing the quality of life in SMA children (*n* = 66) based on demographic and clinical features

Variables	Physical well-being	Psychological well-being	Parent relationship	Social support and peers	Social environment
Gender					
Boys	12.9 ± 2.3	20.6 ± 5.7	24.9 ± 5.0	12.1 ± 4.0	13.0 ± 4.3 *
Girls	12.2 ± 2.1	19.2 ± 5.2	24.8 ± 4.8	13.0 ± 3.6	15.2 ± 3.8
Eductaion of mother					
≤ 12	12.1 ± 2.1*	19.1 ± 5.1 *	23.8 ± 5.0 *	12.1 ± 4.0	13.9 ± 4.1
> 12	13.8 ± 2.1	22.2 ± 6.0	27.6 ± 3.4	13.7 ± 3.1	14.8 ± 3.4
Eductaion of father					
≤ 12	12.3 ± 2.4	18.5 ± 4.9 *	23.4 ± 4.7 *	11.6 ± 4.0 *	13.2 ± 4.3 *
> 12	12.9 ± 1.9	21.8 ± 5.6	26.7 ± 4.5	13.8 ± 3.1	15.4 ± 3.1
Number of children					
< = 2	12.7 ± 2.3	20.0 ± 5.1	25.7 ± 4.8 *	12.4 ± 3.8	13.8 ± 4.0
> 2	12.3 ± 2.2	19.6 ± 6.1	22.9 ± 4.7	12.7 ± 3.8	14.8 ± 3.8
Access to medical facilities					
Yes	12.6 ± 2.3	21.1 ± 5.6 *	24.6 ± 5.1	13.0 ± 3.7	14.3 ± 4.0
No	12.4 ± 2.1	17.2 ± 4.1	25.3 ± 4.6	11.5 ± 3.9	13.8 ± 4.0
Dependence to wheelchair					
No	14.4 ± 2.1 *	20.7 ± 4.1	27.1 ± 3.4 *	12.3 ± 3.7	13.9 ± 3.0
Yes	12.2 ± 2.1	19.7 ± 5.7	24.3 ± 5.0	12.6 ± 3.8	14.2 ± 4.1
Respiratory diseases					
Yes	12.0 ± 2.0	20.1 ± 4.7	23.5 ± 5.7 *	12.5 ± 5.3	13.6 ± 4.7
No	12.7 ± 2.3	19.8 ± 5.6	25.1 ± 4.7	12.5 ± 3.5	14.2 ± 3.8
GI diseases					
Yes	11.6 ± 1.5 *	18.4 ± 4.3	24.1 ± 5.4	10.7 ± 4.7 *	13.5 ± 4.9
No	12.9 ± 2.4	20.5 ± 5.8	25.1 ± 4.7	13.2 ± 3.2	14.4 ± 3.6
History of mood disorders					
Yes	11.9 ± 2.2 *	17.1 ± 5.7 *	23.2 ± 4.9 *	12.4 ± 4.0	14.1 ± 3.7
No	13.0 ± 2.1	21.7 ± 4.5	25.8 ± 4.6	12.6 ± 3.7	14.2 ± 4.1

*GI* gastrointestinal

^*^P-value less than 0.05

## Results

Among 66 children with SMA, there were 12 (35.3%) boys and 22 (64.7%) girls with SMA type II, and there were 19 (59.4%) boys and 13 girls (40.6%) with SMA type III. The mean (SD) age of children with type II SMA was 11.3 (3.5), and it was 15.5 (3.4) in children with type III SMA. Based on SMA type, there was no significant difference between the age of boys and girls.

Table 2 indicates that the health-related quality of life between children with type II and type III SMA is not significant in all 5 subscales, both children's and parent's perspectives.

Table 3 demonstrates that the health-related quality of life in healthy children is significantly higher than in SMA children in all 5 subscales, both children's and parents’ perspective. The highest mean difference was observed in physical and psychological well-being. Moreover, no significant difference was detected between children's and parents’ perspectives in reporting health-related quality of life.

**Table 2:** Comparing the quality of life in children with type II and type III SMA

	Type II SMA (*n* = 34)	Type III SMA (*n* = 32)	*P*-value
Child			
Physical well-being	12.1 ± 2.1	13.0 ± 2.3	0.09
Psychological well-being	19.3 ± 3.0	19.9 ± 2.8	0.42
Parent relationship	24.3 ± 4.9	25.3 ± 4.8	0.43
Social support and peers	12.3 ± 3.9	12.7 ± 3.7	0.67
Social environment	14.1 ± 4.8	14.2 ± 2.8	0.89
Parent			
Physical well-being	12.6 ± 2.1	12.3 ± 2.5	0.67
Psychological well-being	21.2 ± 2.5	20.06 ± 2.7	0.30
Parent relationship	23.7 ± 5.4	24.4 ± 5.2	0.59
Social support and peers	11.4 ± 4.2	12.6 ± 3.6	0.22
Social environment	13.4 ± 4.8	13.9 ± 3.1	0.62

**Table 3:** Comparing the quality of life in SMA children with that of a matched healthy control group

	Case group (*n* = 66)	Control group (*n* = 264)	
	Mean ± SD	Mean ± SD	*p*-value
Child			
Physical well-being	12.6 ± 2.2	19.6 ± 3.9	< 0.001
Psychological well-being	19.6 ± 2.9	25.6 ± 5.7	< 0.001
Parent relationship	24.8 ± 4.9	26.9 ± 5.6	0.005
Social support and peers	12.5 ± 3.8	14.5 ± 3.3	< 0.001
Social environment	14.1 ± 3.9	15.4 ± 3.1	0.004
Parent			
Physical well-being	12.5 ± 2.3	19.4 ± 3.8	< 0.001
Psychological well-being	20.9 ± 2.6	25.9 ± 5.1	< 0.001
Parent relationship	24.7 ± 5.3	27.1 ± 5.0	< 0.001
Social support and peers	12.1 ± 3.9	13.7 ± 3.2	< 0.001
Social environment	13.6 ± 4.1	15.4 ± 2.9	< 0.001

The quality of life in children with SMA was varied according to demographic and clinical features. SMA children whose mothers had less than 12 years of education and also SMA children with history of mood disorders had significant lower score in Physical and psychosocial well-being, and also parent relationships. Low education of fathers also associated with all subscales of the SMA children’s quality of life except physical well-being subscale. Each of demographic and clinical variables is associated with a different subscale of quality of life, details have been shown in Table 1.

## Discussion

In this case–control study, the quality of life was compared between SMA children and a healthy control group, between type II and type III SMA, and also the quality of life was compared according to demographic and clinical features in children with SMA.

The current study indicated that the quality of life in children with SMA varies from that in the matched healthy control group in all subscales. It is important to note that two subscales of quality of life, including physical and psychosocial well-being, were more affected in SMA patients. A systematic review has recently indicated that all subscales are affected by SMA to some degree, however physical well-being is the main domain of life impaired by SMA [23]. Due to the similarity of locomotion impairment on quality of life in neuromuscular disorders, the most common childhood neuromuscular disorder, Duchenne muscular dystrophy (DMD), was considered in the literature review [24]. Most case–control studies have been indicating that the quality of life varies in all subscales [16–18, 25]; however, Zamani et al. [22] observed the low scores in just two subscales, including physical well-being and peers. Opstal et al. [26] observed the low score only in the physical well-being subscale. The differences across studies may be explained by the small sample size and the type of questionnaire used [18], according to a systematic review conducted by Landfeldt et al. [24], 40% of the studies had been done with a small sample size (less than 35).

We did not find any significant difference between the quality of life in children with SMA type II and type III. Since studies have evaluated QoL by different tools [24] and most of them do not report their results according to SMA type, demographic data, or functional ability [19, 20, 27–29], it is hard to draw an appropriate conclusion over the observed differences. However, an inverse association has been suggested by some data [23].

This study showed that girls with SMA had a better score in the social environment subscale in compare to the boys. It may be due to different interests and styles of life in different genders in this age group in Iranian children. However, planning for the promotion of this aspect for the both genders to improve their participation in social activities, including supportive consultation, and providing recreational and educational facilities to change the patient's attitudes is recommended [22].

## Conclusion

The current study declares that the quality of life in children with SMA varies in comparison with healthy control group in all subscales. However physical well-being and psychosocial aspects are the main domains of life impaired by SMA disease. We did not find any significant difference between the quality of life in children with SMA type II and type III. Furthermore, better social score in girls is seen in comparison with boys with SMA patients that may be due to different interests and styles of life in different genders in this age group in Iranian children. At the time of doing this study none of the patients had access to advanced treatments, for sure the QoL after access to new treatment and the multidisciplinary clinic would differ. So, a new study to evaluate and compare the issue of QoL after access to treatment in these patients is recommended.

### Strengths and limitations

Compared to many studies, the strength of our study was comparing the quality of life according to SMA type and some demographic and clinical features. Moreover, the design of our study was case control, which provides more reliable results compared to cross-sectional studies. However, we did not have adequate sample size to analyze our data based on functional status (non-sitter, sitter, walkers).

## Data Availability

The datasets used and/or analysed during the current study are available from the corresponding author on reasonable request.

## Abbreviations

SMA: Spinal Muscular Atrophy
HRQoL: Health-related quality of life
QoL: Quality of life
DMD: Duchenne muscular dystrophy
